# Extracellular Vesicles in Hematological Malignancies: From Biomarkers to Therapeutic Tools

**DOI:** 10.3390/diagnostics10121065

**Published:** 2020-12-09

**Authors:** Jihane Khalife, James F. Sanchez, Flavia Pichiorri

**Affiliations:** 1 Judy and Bernard Briskin Center for Multiple Myeloma Research, City of Hope, Duarte, CA 91010, USA; jihane.khalife@gmail.com (J.K.); jamsanchez@coh.org (J.F.S.); 2 Department of Hematologic Malignancies Translational Science, City of Hope, Duarte, CA 91010, USA

**Keywords:** exosomes, biomarkers, leukemia, lymphoma, multiple myeloma

## Abstract

Small extracellular vesicles (EVs) are a heterogenous group of lipid particles released by all cell types in physiological and pathological states. In hematological malignancies, tumor-derived EVs are critical players in mediating intercellular communications through the transfer of genetic materials and proteins between neoplastic cells themselves and to several components of the bone marrow microenvironment, rendering the latter a “stronger” niche supporting cancer cell proliferation, drug resistance, and escape from immune surveillance. In this context, the molecular cargoes of tumor-derived EVs reflect the nature and status of the cells of origin, making them specific therapeutic targets. Another important characteristic of EVs in hematological malignancies is their use as a potential “liquid biopsy” because of their high abundance in biofluids and their ability to protect their molecular cargoes from nuclease and protease degradation. Liquid biopsies are non-invasive blood tests that provide a molecular profiling clinical tool as an alternative method of disease stratification, especially in cancer patients where solid biopsies have limited accessibility. They offer accurate diagnoses and identify specific biomarkers for monitoring of disease progression and response to treatment. In this review, we will focus on the role of EVs in the most prevalent hematological malignancies, particularly on their prospective use as biomarkers in the context of liquid biopsies, as well as their molecular signature that identifies them as specific therapeutic targets for inhibiting cancer progression. We will also highlight their roles in modulating the immune response by acting as both immunosuppressors and activators of anti-tumor immunity.

## 1. Introduction

EVs are lipid bilayer structures ranging in size from 15 to 10,000 nm, are released from almost all cells (normal and cancerous) present in body fluids, and they are considered as delivery vehicles responsible for local and distant cellular communication [1]. In the late 1960s, the role of EVs was thought to be limited to the export of cellular waste products, first discovered during the programmed enucleation of maturating red cells in sheep [2]. Since then, considerable efforts were made to study the biology of EVs, and today they are becoming a major focus as regulatory molecules in health and disease. In cancer, tumor-derived EVs have been implicated in cell–cell communication and the transfer of information between malignant cells and cells of the microenvironment [3]. They carry molecules that are completely different from those released by EVs originating form healthy counterparts [4]. Therefore, tumor-derived EV cargo molecules (also known as bioactive molecules carried by EVs and delivered to the surrounding cells) are considered unique markers, and are released by tumors to increase proliferation, metastasis [5], and immune escape by remodeling the microenvironment in favor of disease progression [6]. In hematological malignancies where the cancerous cells generated from blood-forming tissues are highly heterogenous, there is a lack of sufficient monitoring tools for adequate diagnosis and treatment [7]. Therefore, novel non-invasive clinical approaches are being developed for proper diagnosis and monitoring of disease progression and response to treatment [8]. EVs are considered strong, minimally invasive diagnostic biomarkers for malignancies because of their capacity to carry biologically active molecules that reflect the cell of origin and their ability to protect their cargoes from nucleases and protease activity. They were extensively studied in solid tumors [9,10]; however, in hematological malignancies, they are in the process of being defined. In this review, we will focus on the most recent findings discussing the role of EVs as diagnostic markers and biomarkers of disease progression and drug resistance in the most prevalent hematological malignancies, as well as their involvement in the cross-talk between malignant cells and the bone marrow (BM) microenvironment, making them strong therapeutic targets. We will also discuss their dual effects in immune escape and anti-tumor immunity.

## 2. Extracellular Vesicles: Biogenesis, Composition, and Cellular Uptake

EVs are classified by four increasing orders of size and the mechanism of biogenesis: (i) Exosomes (30–150 nm); (ii) microvesicles or ectosomes (50–1000 nm); (iii) large vesicles (>1000 nm); and (iv) apoptotic bodies (>1000 nm) [11].

- The biogenesis of exosomes, known as the smallest EVs, starts by inward cleavage of the plasma membrane to form early endosomes. Endosomes then undergo further maturation to form intraluminal vesicles. Those vesicles, through RAB27- and VPS33b-dependent mechanisms, evade lysosomal degradation and fuse to the plasma membrane to release exosomes [12].
- Microvesicles or ectosomes do not use the endosomal pathway for their formation; instead, they are formed by a pathway that involves calcium influx and release of cargoes by remodeling the cortical cytoskeleton. Unlike exosomes, they are not formed in a consistent manner, but are dependent on specific cell types, such as neutrophils [13,14].
- Large vesicles are formed from cleavage of the cytoplasmic portion of intact living cells, reach up to 10,000 nm in size, and contain cytoskeletal structures. Large vesicles are only formed by specific cells, such as B lymphoblastic and prostate cancer cells [15,16].
- Apoptotic bodies’ biogenesis occurs during apoptosis or programmed cell death. They are formed when apoptotic cells’ cytoplasms and plasma membranes begin to break into fragments. They contain both cytosolic components and nuclear fragments. They are eliminated through phagocytosis by surrounding cells [17].

Circulating vesicles are mainly composed of exosomes and microvesicles, which are, to date, the only types of vesicles that have shown to play a role in short- or long-distance cell-to-cell communication. Despite their overlap in size and the inability of current purification methods to discriminate between them, exosomes and microvesicles can contain similar molecular information and be released by similar cell types [13]. However, their composition is strictly dependent on the cell type from which the vesicles originated, and not the vesicles’ type or size. Molecular cargoes, once enveloped in EVs, are protected from extracellular enzymes and can be delivered to recipient cells intact, enabling specific molecular functions [18].

Genomic and proteomic analyses show that the molecular cargoes of EV consist of mRNAs, microRNAs, and plasmid DNA. Although both exosomes and microvesicles can encapsulate mRNAs and microRNAs, microvesicles can only carry plasmid DNA. EV-encapsulated mRNAs can be translated to proteins by the recipient cells [19,20]. Other than mRNAs and microRNAs, EVs can also carry small, non-coding RNA, such as tRNAs and small interfering RNA (siRNA), among others [21]. Depending on the nature of the cell the EVs originated from, their molecular content could particularly include proteins associated with lipid rafts, such as sphingomyelin, tetraspanins, and heat shock proteins [22].

Although not extensively proved, it has been reported that EVs carry particular bioactive molecules packaged to be delivered to pre-designed target cells, such as was observed in breast cancer, where EVs released by fibroblasts are taken up selectively by cancer cells, promoting their migration, activity, and metastasis [23]. However, how EVs target pre-designed cell types within the hematopoietic niche is starting to be defined. A study showed that tetraspanins, the most common type of protein associated with EVs, play a major role in targeting vesicles to selective cell types. For instance, tetraspanins such as CD37, CD53, and TSSC6 have been exclusively found in hematopoietic cells, and were shown to interact with specific target cells expressing MHCI/II or T-cell/NK-cell costimulatory CD2, among others. For example, if a regulatory signal is sent from a hematopoietic cell to a T or NK cell, EVs released from the parental cell would be selectively enriched in CD37, CD53, or TSSC6 tetraspanins [24].

Once released from the parental cell, EVs can arrive at multiple destinations; they can release their content in the surrounding interstitial space or biofluids (urine, blood), or they can be taken up by recipient cells at short or long distances. Cellular uptake is the “safest” route for EVs, as they appear to have a short half-life in biofluids, especially in circulating blood, where their half-life does not exceed two minutes before being redistributed into cells [25]. EVs are delivered to target cells by several mechanisms, including membrane fusion, phagocytosis, receptor-mediated caveolin-clathrin, or endocytosis mediated by lipid rafts. All those mechanisms will lead to entry of the EVs to the intracellular compartment, where they deposit their bioactive cargoes [26]. RNA (mRNA, microRNA, siRNA) cargoes are deposited in the endoplasmic reticulum in which they exert their functions, whereas empty EVs stall at the endoplasmic reticulum and fuse with lysosomes for degradation [27]. 

EV secretion occurs under both physiological conditions and stress response. However, cells under stress release significantly higher amounts of EVs than resting cells. Hypoxia is believed to be one of the major stress pathways that favor EV release. Hence, tumor cells secrete almost 60-fold higher quantities of EVs in the plasma than their healthy counterparts. Secreted EVs contain distinctive molecular cargoes that are mostly associated with tumor growth and immune suppression. Selective cargoes play an essential role in tumor development and progression [28,29]. This finding indicates that tumor-derived EVs could serve as critical biomarkers for cancer and could be considered as specific therapeutic targets.

### 2.1. Potential Roles of Circulating EVs as Biomarkers and Therapeutic Targets

#### 2.1.1. Acute Myeloid Leukemia

Acute myeloid leukemia (AML) is the most common acute leukemia in adults and is characterized by clonal expansion of abnormal myeloid precursors in the bone marrow, named “blasts”, that interfere with the normal hematopoiesis of blood cells. Symptoms of AML include fever, anemia, shortness of breath, bleeding, and increased risk of infections. Although considerable efforts have been made in the past few years, such that 45% of patients who attain complete remission can be expected to survive three or more years, AML is still the most incurable hematological malignancy, especially in older patients (>60 years), where the five-year survival rate is still less than 25% [30].

Tumor-derived EVs (TEV) play an important role in AML. They carry important AML diagnostic markers, such as NPM1 and FLT3-ITD mRNAs [31]. Other than their role in diagnosis, TEVs were demonstrated to participate in AML tumorigenesis. Newly diagnosed AML patients showed a significantly higher percent of EVs released in their plasma in comparison to healthy controls [28]. EVs have been essentially implicated in the transformation of the myeloid cell environment into a leukemia-favoring space by either an autocrine or paracrine crosstalk. Indeed, one recent study showed that apoptosis-resistant protein cargoes (MCL-1, BCL-2, BCL-XL) were transported via EVs in a paracrine manner between AML blasts and elicited therapy resistance in recipient cells [32]. TEVs also contributed to intercellular communication between AML cells and cells of the bone marrow microenvironment. AML-derived EVs upregulated the hematopoiesis suppressor factor DKK1 in bone marrow stromal cells (BMSC) and reduced their ability to support normal hematopoiesis and osteogenesis in vivo [33]. They also altered the proliferation and migration responses of co-cultured stromal and hematopoietic progenitor cells by transferring CXCR4 and IGF-IR mRNA to the recipient cells, helping to remodel the microenvironmental niche in favor of leukemic cell growth [31]. Taken together, these findings highlight the unique feature of circulating EVs—to contain cargoes, representing a contribution from leukemic cells or cells of the tumor microenvironment that are sources of pro-tumorigenic signals. This characteristic makes serum EVs a robust biomarker platform for the monitoring of AML development.

In newly diagnosed AML patients, serum EVs were enriched in myeloblastic markers important for AML diagnosis (CD33, CD34, and CD117), as well as in essential proteins, such as genes related to MHC class I and TGFβ1 [34]. EV-derived TGFβ1 was of particular interest, as levels were dramatically high in a large majority of AML patients. EV-TGFβ1 levels have been shown to inversely correlate with response to chemotherapy. After a course of induction chemotherapy, a significant reduction in plasma EV-TGFβ1 protein levels occurred concomitant with the reduction of blasts in the bone marrows. Patients in long-term complete remission had low EV-TGFβ1, similarly to those seen in the plasma of healthy controls. These data indicate the significance of plasma EV-TGFβ1 levels as an indirect measure of leukemic blasts in the bone marrow. Changes in EV-TGFβ1 levels could be a potential diagnostic marker to stratify AML patients into predicted early, late, and complete remission, as well as relapse status [35]. At the immunological level, EV-TGFβ1 showed to play a potent immunosuppressive role in natural killer (NK) cytotoxicity. EV-TGFβ1 in the sera of AML patients suppressed NK-cell activity by targeting the activating receptor NKG2D [34]. Other than its role as a biomarker, EV-TGFβ1 could be a potential therapeutic target to reinitiate NK-cell immune function in AML.

Another potential biomarker of myeloid tumors are EVs enriched in CD13 (a mature, undifferentiated myeloid surface marker). Circulating EVs containing CD13 were detected in the sera of patients with MPN (myeloproliferative neoplasm, which could evolve to AML) and at a higher level in those with myelodysplastic syndrome (MDS) as compared to that in normal subjects. Higher-risk MDS is associated with the highest amount of EV-CD13, with respect to patients with low risk MDS or patients with MPN, and has levels comparable to those detected in AML [36]. Taken together, these data showed that EV-CD13 could be considered a robust prognostic marker of myeloid tumor progression and might correlate with evolution toward leukemia. More specifically, EV concentration was shown to correlate with essential thrombocythemia (ET), a major symptom of MPN that, over the long term, may progress to myelofibrosis, AML, or MDS [37]. For instance, EVs enriched in platelet activation and prothrombotic factors, as well as an adhesion molecule marker, were observed in the sera of patients with ET. Increased expression of adhesion molecules on endothelial cell walls facilitate platelets’ and leukocytes’ interaction with endothelial cells, a phenomenon that is known to trigger thrombotic diathesis in ET [38,39]. Interestingly, the levels of EVs carrying endoglin, a key endothelial marker highly expressed in neoangiogenesis, were abundantly released in patients’ sera compared to that in healthy controls. EV-endoglin levels were significantly reduced in patients treated with an anti-platelet agent, supporting the role of endothelial cell damage in the pathogenesis of ET [39]. These findings highlight the importance of circulating EVs as markers of reactive thrombocythemia where specific disease markers are still lacking.

Not only do EVs carry mRNA and protein cargoes that serve as biomarkers, but they can also transfer microRNAs, active molecules that are well-established as biomarkers. However, EV-microRNAs appear to be an even more selective non-invasive biomarker platform over circulating microRNAs. EV cargoes load selective microRNAs representative of cells of origin (blast cells or cells of the leukemic niche) and have a functional role in tumorigenesis. The resulting highly restricted vesicle-microRNA cargoes offer a selective biomarker profile over that of circulating, more diverse microRNAs, which are often complexed with lipoproteins [40]. AML cell-derived EVs have been shown to contain a characteristic microRNA profile. Higher serum EV let-*7a*, miR-*99b*, -*146a*, -*191*, -*1246*, -*10b*, and -*155* concentrations were detected when compared to healthy subjects, and some of those EV-microRNAs predicted poor prognosis [40,41,42]. EV-miR-*155* was of particular interest as a selective and sensitive biomarker for detection and monitoring of AML for several reasons: (i) First, high EV-miR-*155* levels were redundantly detected not only in AML patients [42], but also in AML models at serum concentrations, reaching up to 1000-fold above cellular levels. Using a single exosomal microRNA score to separate leukemic cells from controls, EV-miR-*155* independently correlated with AML burdens [40]. (ii) Secondly, EV-miR-*155* loads not only distinguished patients from healthy subjects, but their levels also proportionally coincided with the stages of disease. Their amounts were directly correlated with white blood counts and complex karyotypes [42]. EVs containing miR-*155* (EV-miR-*155*) were also detected in MPN patients at lower levels than in AML, but had functional activity. They induced an increase in colony-forming unit numbers when incorporated into CD34+ neoplastic progenitor cells [43]. (iii) Third and most importantly, both leukemic blasts and BMSCs contributed to serum EV-miR-*155* release, further supporting EV-miR-*155* as an indicator of AML. On one hand, BMSC-derived EVs enriched in miR-*155* communicated with AML blasts and protected them from tyrosine kinase inhibitor (TKI) treatment. In recipient cells, miR-*155* induced a downregulation in apoptotic and cell differentiation genes, the cause of TKI resistance [44]. On the other hand, EV-miR-*155* derived from AML blasts disrupted the normal hematopoietic niche. They suppressed residual healthy hematopoietic stem progenitor cell (HSPC) function by decreasing their clonogenicity. MiR-*155* mediated suppression of c-MYB, a transcription factor involved in HSPC differentiation and proliferation [45]. Together, these findings confirm that serum EV-miR-*155* should be considered clinically—not only as an early prognostic marker and a monitor of disease progression, but also as a selective biomarker of drug resistance and a potential therapeutic target in AML.

#### 2.1.2. Chronic Myeloid Leukemia

Chronic myeloid leukemia (CML) is a myeloproliferative leukemia with slow-growing blasts in the bone marrow, characterized by polycythemia vera, myelofibrosis, and thrombocythemia. Among CML patients, 95% have a distinctive cytogenetic abnormality, the Philadelphia chromosome (Ph1), which results in a fused *BCR/ABL* gene and in the production of an abnormal tyrosine kinase protein that causes the disease. Those patients are often treated with the TKI imatinib. Most of them respond well to the treatment, with a median survival time that can approach normal life expectancy. However, the remaining ~5% of CML patients who are Ph1-negative respond poorly to the treatment, and have shorter survival than Ph1+ patients [46].

In CML, research studies on EVs are not as elaborated as in AML. However, Ph1+ CML-derived EVs were shown to be highly enriched in BCR-ABL mRNA. Because they carry the chromosomal abnormality present in ~95% of CML patients, EVs themselves could serve as an alternative early diagnostic marker for detection of Ph1+ CML instead of the labor-intensive conventional fluorescent in situ hybridization technique performed on bone marrow aspirates to detect *BCR/ABL* translocation [46,47]. Moreover, cell–cell communication via TEVs has shown to promote CML malignancy. Horizontal transfer of EV-BCR-ABL mRNA from leukemic to normal mononuclear cells induced malignant transformation of the latter by mechanism of genomic instability that led to DNA breakage and recombination [47]. BCR-ABL mRNA was not only transferred to mononuclear cells, but also to immune cells of the microenvironment. Cai et al. found that CML-derived EVs carrying BCR-ABL interacted with normal neutrophils, causing their malignant transformation and reduction of their phagocytic activity. K562-EV injection into mice caused several symptoms of CML, including splenomegaly. De novo BCR-ABL mRNA and protein synthesis caused the development of the disease in vivo [48]. Taken together, these data highlight that EVs enriched in BCR-ABL induced neoplastic cell growth and an immuno-suppressive environment promoting leukemogenesis. 

Similarly to AML, CML-derived EVs were enriched in TGF-β1 protein. However, in CML, EVs containing TGFβ1 did not induce an immunosuppressive environment; instead, they promoted proliferation and survival of leukemic cells in an autocrine manner, both in vitro and in vivo. The proposed mechanism involved a ligand–receptor interaction between TGF-β1 found in CML-derived exosomes and the TGF-β1 receptor in CML-recipient cells, causing an increase in anti-apoptotic molecules BCL-w, BCL-xl, and survivin, and reduction of the pro-apoptotic proteins BAD, BAX, and PUMA [49].

Interaction between BMSCs and CML cells also play a key role in CML pathogenesis. EVs have contributed to the intercellular communication between these two cell compartments. CML-derived EVs stimulated BMSC to produce the IL-8 chemokine, which in turn promoted leukemic cell survival [50]. They were also able to malignantly transform healthy mesenchymal stromal cells (MSC) and endothelial cells [51,52]. CML-derived EV uptake by endothelial cells resulted in enhanced angiogenesis via delivery of an onco-microRNA, miR-*92a*, known to regulate endothelial gene expression. In recipient human umbilical vein endothelial cells, miR-*92a* directly targets integrin α5 mRNA, an important factor that negatively regulates endothelial cell growth [52]. These data suggest that exosomal miR-*92a* plays an important role in neoplasia-to-microenvironmental cell communication in CML and may be targeted therapeutically.

One of the major challenges faced during treatment of Ph1+ CML patients is that around 40% of them maintain an undetectable minimal residual disease (UMRD) upon imatinib discontinuation. In a search for possible biomarkers that could select Ph1+ CML patients with UMRD who discontinued imatinib, Ohyashiki and coworkers found that EV-microRNAs in plasma were the most promising factors. EV-microRNA profiling was conducted in patients with CML who discontinued imatinib therapy but still have UMRD. Among the several EV-microRNAs tested, significantly low levels of EV-miR-*215* were detected in patients’ plasma with respect to those of healthy controls or patients with UMRD who had continued imatinib therapy [53]. Circulating EVs carrying the tumor suppressor miR-*215* could play an important role in selecting CML patients who achieved treatment-free remission.

#### 2.1.3. Chronic Lymphocytic Leukemia

Chronic lymphocytic leukemia (CLL) is a cancer of lymphoid cell origin characterized by progressive accumulation in the bone marrow of immature lymphocytes, which are usually monoclonal in origin. CLL is one of the most common types of leukemia in adults, and typically progresses more slowly than others. Its manifestations resemble that of B-cell neoplasms. At early stages of the disease, there are no typical symptoms. Later, non-painful lymph node swelling, fatigue, easy bruising or bleeding, and petechiae may occur. CLL confers a higher survival rate than other leukemias. However, although patients may have a complete or partial response to initial therapy, disease relapse occurs frequently [54].

CLL is the hematological malignancy whose pathogenesis is most stringently associated with a tumor-supportive microenvironment. CLL cells are heavily dependent on their microenvironment for survival. EVs have emerged as a major mechanism of intercellular communication that creates a favorable environment for promoting CLL progression. CLL-derived EV levels were significantly higher compared with those in healthy subjects. They were shown to interact with and modulate BMSCs. More specifically, CLL cell-derived EVs activated the AKT/mammalian target of rapamycin/p70S6K/hypoxia-inducible factor-1 alpha axis in CLL-BMSC, which resulted in excessive production of the vascular endothelial growth factor, a survival factor for CLL-B cells [55]. CLL cells communicate with several compartments of the microenvironment. Other than BMSC, they interact with endothelial cells via EVs, inducing an inflammatory phenotype that resembles the phenotype of cancer-associated fibroblasts. CLL-derived EVs rapidly activated kinases p-Akt, p-ERK1/2, and NF-κB pathways in target cells. As a result, stromal and endothelial cells showed enhanced proliferation, migration, and secretion of tumorigenic cytokines/chemokines (IL-6 and IL-8) and proangiogenic factors, thus providing a nurturing environment for CLL cells [56]. Moreover, EVs derived from bone marrow mesenchymal stromal cells induced a decrease of leukemic cell spontaneous apoptosis and an increase in their chemoresistance to several drugs, including fludarabine, ibrutinib, idelalisib, and venetoclax. In this context, an EV microarray study showed elevated expression of key oncogenes involved in the B-cell receptor pathway, such as CCL3/4, EGR1/2/3, and Myc [57]. Specific targeting of EVs involved in a bidirectional cross-talk between CLL cells and the bone marrow microenvironment, particularly those containing Myc, would be of great therapeutic interest to impede CLL cell growth, progression, and resistance to therapy.

EV protein cargoes carrying CD markers have shown to serve as predictive biomarkers in CLL patients at several stages of the disease. An antibody microarray profiling of the membrane protein content of plasma EVs revealed elevated levels of CD5, CD19, CD31, CD44, CD55, CD62L, CD82, HLA-A, HLA-B, HLA-C, and HLA-DR and low levels of CD21, CD49c, and CD63, whereas none of these proteins were detected on EVs from the plasma of healthy individuals [58]. More specifically, plasma EVs containing CD19 and CD37 were highly elevated at advanced stages of the disease and proportionally correlated with a high tumor burden [59]. Another recent study showed that plasma EVs containing CD52 (a mature lymphocytic marker generated by CLL-B cells) can be a specific diagnostic signature for CLL progression. Plasma CD52+ EV levels dropped significantly after therapy and remained at low levels in some patients, whereas an increased accumulation of EV-CD52 was detected in post-therapy patients in which the disease progressed and required therapy for a second time [60].

The B-cell receptor (BCR) signaling pathway plays an important pathogenic role in CLL. Mutations of BCR pathway components that lead to constant activation of downstream kinases are often present. BCR-associated kinase inhibitors, such as ibrutinib (BTK inhibitor), contributed to improved clinical therapy of CLL in recent years. MicroRNA profiling of plasma-derived EV identified a distinct EV microRNA signature, in part associated with BCR activation. Interestingly, expression of miR-*150* and miR-*155* in EVs increased proportionally with BCR activation [61]. Serum EV miR-*150* and miR-*155* could play a promising diagnostic marker for patients with an activated BCR pathway who could potentially benefit from treatment with inhibitors targeting BCR-associated kinases. Additionally, serum EV-miR-*155* has been shown to play a useful marker of disease progression from monoclonal B-cell lymphocytosis (MBL) to CLL. Ferrajoli et al. found increased levels of miR-*155* in circulating EV in individuals with MBL, and still higher levels in B cells from patients with CLL, when compared with B cells from normal individuals [62]. Plasma levels of EV-miR-*155* could be useful biomarkers for the risk of progression from MBL to CLL.

Some CLL patients who become resistant to multidrug therapy might develop a clinical syndrome entitled Richter syndrome (RS), which is defined as the transformation of CLL into an aggressive lymphoma, most commonly, diffuse large B-cell lymphoma. Treatment of RS is usually difficult and relies only on conventional chemotherapy. Identification of biomarkers in those patients would be of great benefit to address the disease at its early stages. A recent study identified a microRNA signature that could predict the evolution of therapy-resistant CLL to RS. miR-*19b* in EVs showed tumor-supportive effects and were specifically upregulated in the sera of CLL patients that transitioned to RS [63]. Monitoring serum EV-miR-*19b* levels would be a useful clinical tool to identify CLL patients that evolved to RS before showing severe symptoms.

#### 2.1.4. Multiple Myeloma

Multiple Myeloma (MM) is a cancer of terminally differentiated plasma cells (PCs) that involves multiple sites within the BM. It is diagnosed by the presence of higher than 10% of monoclonal long-lived PCs within the BM and bony/extra-medullary plasmacytoma. Patients are diagnosed by the presence of high proportions of monoclonal free immunoglobulin in their sera, called paraprotein. Symptoms include anemia, renal failure, proteinuria, and osteolytic bone lesions. MM represents the second most commonly diagnosed hematological malignancy worldwide. It is highly treatable but rarely curable. The presence of multisite tumor infiltrates, each with differing degrees of drug sensitivity, makes the disease highly heterogenous and variable in survival, ranging from few weeks to more than 10 years with frequent episodes of remission and relapse [64].

The most influential interactions that highly contribute to MM pathogenesis exist between MM-PCs and BMSCs/MSCs of the microenvironment. EVs are among the main factors that play a role in such cross-talk. Of note, the content of EVs released by BM MSCs and BMSCs differ significantly between MM patients and healthy individuals. Whereas EVs derived from MM-BMSCs promote MM tumor growth, BMSC-EVs in noncancerous individuals inhibited the growth of MM cells. Mechanistically, MM-BMSCs transferred EVs containing low levels of the tumor suppressor miR-*15a* and high levels of IL6 and CCL2 into MM cells, inducing their proliferation and survival [65]. In another study, Yaccoby et al. showed that MSCs from healthy individuals acquired the tumor phenotype of MSCs from MM patients following co-culture with MM cells [66]. These findings give rise to the question of whether MM-PC cells are effectively the first initiators of cross-talk within the BM microenvironment. Indeed, in a recent study, we showed that MM-PCs carrying chromosome 13 deletion (Del13) communicated with the BM microenvironment via EVs and changed its oncogenic landscape. Specifically, we have shown that the tumor suppressor miR-*16* (miR-*15a*/*16–1* cluster) was carried by EVs from MM cells, and its loss was associated with Del13. MM-derived EVs carrying low levels of miR-*16* strongly differentiated monocytes to M2-tumor supportive macrophages, known as important components of the tumor microenvironment. However, EVs isolated from PCs without the chromosomal aberration did not affect monocyte differentiation. Mechanistically, we showed that miR-*16* directly targeted and inhibited the IKKα/β complex of the NF-κB canonical pathway, which is critical not only in supporting MM cell growth, but also in polarizing macrophages toward an M2 tumor-supportive phenotype [67].

Despite the difficulty in clearly tracking the original cell type that released EVs in question (whether stromal or MM-PCs) to initiate a mutual communication between MM cells and the BM microenvironment, taken together, the aforementioned findings clearly indicate that in MM, EVs carry low levels of the tumor suppressors miR-*15a* and miR-*16* that are both present in the same gene cluster, and intervening in the EV-mediated BM microenvironment interactions may be a valuable therapeutic tool to block the communication between MM cells and the microenvironment, thus impeding MM cell survival. Similar to the exosome modulation technique used to formulate TRAIL-decorated EVs that showed promising anti-tumor effects when either injected at the tumor sites or in a systemic manner [68], a formulation of EVs armed with miR-*15a* and-miR-*16* would be of useful clinical application to improve long-lasting anti-MM therapies.

In a recent study, Deng et al. have demonstrated that the long non-coding RNA LNC00461, a sponge for miR-*15a*/*16*, was highly expressed in MM exosomes and enhanced MM cell proliferation by relieving the inhibitory effect of (miR)-*15a*/miR-*16* on the anti-apoptotic protein BCL-2. Knockdown of LINC00461 dramatically reduced MM cell proliferation and induced cell apoptosis [69]. Targeting EV-LNC00461 either in circulation or at tumor sites may also be another therapeutic application to indirectly revert the expression of both miR-*15a* and miR-*16* in MM.

Another exosomal microRNA that may serve as a specific therapeutic target to hinder the mutual communication between MM and stromal cells is EV-miR-*146a*. A microarray profile performed on EV-microRNAs derived from MM cells and transferred to MSCs showed that, among the 19 EV-microRNAs significantly dysregulated, EV-miR-*146a* was the most highly upregulated. miR-*146a* induced an increased secretion of several tumorigenic cytokines/chemokines by MSCs, including CXCL1, IL-6, IL-8, IP-10, MCP-1, and CCL-5 in a NOTCH signaling-dependent manner. In turn, deregulation of the chemokine/cytokine profile stimulated MM cell survival and pro-tumoral activity [70].

Monitoring MM progression in patients is an essential step to adequately choose the most appropriate therapy. Because of a comparative lack of appropriate monitoring tools with respect to other malignancies, there is an urgent need for novel non-invasive prognostic tools applicable in the clinic to easily differentiate MM from other PC neoplasia and to predict the disease stage and treatment response [71]. In this context, several studies have shown that EVs released in the sera of MM patients could serve as especially useful non-invasive prognostic biomarkers. More specifically, EVs carrying microRNAs were the most predominant small RNAs differentially expressed in the peripheral blood of MM patients and healthy controls. A small RNA sequencing of circulating EVs revealed two miRNAs, let-*7b*, and miR-*18a* to be the most significantly correlated with negative progression-free survival and overall survival. This aspect is of significant importance, since from a large exosomal miRNA panel, only EV-let-*7b* and EV-miR-*18a* showed a prognostic association with patient survival [72]. Monitoring EV-let-*7b* and EV-miR-*18a* in circulation may constitute a beneficial prognostic and risk stratification tool for patients with MM.

Other than its therapeutic role in blocking communication between MM cells and the BM microenvironment, we have shown that extracellular miR-*16* is among the few microRNAs that can be used as a prognostic biomarker in newly diagnosed MM patients treated with proteasome inhibitor-based therapy. MM patients with high levels of circulating miR-*16* had longer overall survival with respect to patients with low levels of miR-*16* in their sera [73]. In agreement with our findings, an exosome-associated miRNA expression pattern performed on patients resistant to bortezomib (proteasome inhibitor) revealed that exosomal miR-*16*, miR-*15a*, miR-*20a*, and miR-*17* were strongly downregulated in circulation, and were associated with drug resistance [74]. Considering that a high proportion of patients have innate or developed resistance to bortezomib during the course of treatment, and that routine workups of MM reveal little information in this regard, the aforementioned panel of circulating EV-miRNAs could be used as predictive biomarkers for bortezomib resistance. Such a prognostic panel of bortezomib resistance is of great importance and should be clinically applicable for improved personalized therapy.

Circulating MM EV cargoes other than microRNAs were also reported as potential biomarkers of the disease. EV isolated from the sera of MM patients were highly enriched in tumor-related CD markers CD38, CD138, and CD44 [39,75,76]. EV-CD38 positively correlated with the MM clinical international staging system. Other than being a cellular marker, CD38 is reported to function as an ectoenzyme. It converts the adenosine precursor (ATP or NAD+) to adenosine. Adenosine, a known potent immunosuppressor, plays a critical role in tumor formation in the BM niche [77]. Because BM levels of adenosine are higher in MM patients compared to those in patients with monoclonal gammopathy of undetermined significance (MGUS) or smoldering myeloma (SMM), EVs containing CD38, or similar ectoenzymes (CD39 and CD73) highly released in circulation [77], might be used as potential screening markers for differentiation of these conditions, as well as the different stages of MM.

MM-derived EVs carrying CD138 (marker of mature PC) were also detected in the plasma of MM patients, and their amounts directly correlated with disease state and tumor burden. Patients with progressive disease had a significant spike in serum EV-CD138 as compared to those in partial or complete remission. Plasma EV-CD138 numbers could sensitively predict the transition between remission and progressive disease in the absence of clinically used markers of relapse in individual patients [75]. Along with these findings, using EV proteomic profiling, we have identified phagocytic glycoprotein-1 (CD44) as a novel associated marker that inversely correlated with overall survival. MM patients with increased risk of death were associated with increased CD44 in their sera [76]. EV-CD44 is another tumor-related antigen that can be used as a prognostic biomarker in MM.

From the same EV proteomic profiling analysis, we also found the MHC-I antigen-presenting as complex, and its binding protein β2-Microglobulin (β2-MG) to be highly enriched in EVs of MM patients’ sera and BM aspirates. These findings are of high significance because of the known increased proteasome activity in MM as compared to other cancers, and the ability of functional MHC-I to bind and present peptides, generated from protein degradation by the proteasome [76]. β2-MG has also been shown to play a pivotal role in the tumor microenvironment by inducing cancer cell protection from macrophage phagocytosis [78]. Targeting EV-β2MG would be of significant interest to reactivate immune function in the MM BM niche.

The treatment of MM has been substantially improved over the past two decades because of the incorporation of novel therapeutic approaches. Despite the introduction of immunomodulatory drugs and proteasome inhibitors, there is no confirmed curative approach, and autologous bone marrow transplantation remains the standard of care for young or healthy elderly patients with newly diagnosed MM. However, allogeneic bone marrow transplantation has been limited by high mortality rates, primarily from graft-versus-host disease (GVHD). Monitoring GVHD would be of great importance in this context. EVs have been shown as potential biomarkers of GVHD following BM allograft transplantation. In particular, CD46- and CD26-positive EVs were associated with an increased risk of developing GVHD, whereas CD31- and CD106-positive EVs were correlated with a reduced risk of this complication [79]. These biomarkers could be of significant use for predicting the onset of GVHD in MM.

As mentioned earlier, a therapeutic response in MM is highly unpredictable, and the survival rate can be variable among patients. One reason for such a phenomenon is that some patients develop multidrug resistance (MDR) and go into relapse. Currently, there are no clinical procedures that can continuously monitor MDR. In a recent comprehensive study, Krishnan et al. developed a liquid biopsy to predict patients’ unresponsiveness to treatment. The liquid biopsy consisted of distinct EV populations in particular microparticles (MP) containing cargoes that can be used as prognostic markers for MM stages and MDR. Confirming other reports, CD138+ circulating MP elevated gradually with disease progression, whereas a specific MP population consisting of stem cell markers and P-glycoproteins (CD138-P-gp+CD34+) was seen to be significantly elevated in the plasma of patients with aggressive MDR disease [80]. MP enriched with CD138-P-gp+CD34+ cells is the first screening marker reported so far to monitor MDR and possible relapse in MM, and could be potentially used in clinics to support the routine clinical workflows.

#### 2.1.5. Other Hematological Malignancies

Circulating EVs serve as prognostic markers and therapeutic targets in other hematological malignancies as well. Studies have shown that serum EV levels were drastically high in Hodgkin’s lymphoma (HL), non-Hodgkin’s lymphoma (NHL), and Waldenstrom’s macroglobulinemia compared to disease-free control subjects [39]. Circulating EVs have been proposed as new molecular markers for differentiating between HL and NHL. The tumor-related antigen content of lymphoma cell-derived EVs (LCEV) correlated with lymphoma subtypes. LCEV released in the sera of patients with NHL were enriched in CD19 and CD20 [39], while EVs isolated from patients with HL were enriched in the tumor-related antigen CD30, also known as tumor necrosis factor receptor 8 (TNFR8). The latter has been significantly correlated with unfavorable outcomes [81]. EVs derived from CD20+ lymphoma cells were of particular interest in NHL and were considered feasible prognostic markers for disease progression and antibody-based therapy, as their circulating levels directly correlated with CD20+ lymphoma cells. A recent study has shown that CD20+ LCEV can capture rituximab, the first approved anti-CD20 antibody drug. In patients with progressive disease, the high levels of circulating CD20+ EVs played the role of “decoy”. They were demonstrated to directly interact with rituximab and inhibit its therapeutic anti-cancer effect, thus rendering patients resistant to anti-CD20 antibody treatment [82]. In such cases, removal of circulating EVs by either neutralizing them or inhibiting their biogenesis may be a favorable therapeutic personalized approach to re-sensitize NHL patients to anti-CD20 antibody therapy. For instance, in an EL4 lymphoma model, attenuation of EV biogenesis and release by use of dimethyl amiloride (an inhibitor of Na+/Ca2+ exchange) was shown to inhibit tumor growth [83]. 

In classical HL, EVs enriched in CD30 (TNFR8) was not only an important biomarker of the disease, but also showed functional pro-tumoral effects. They facilitate the communication between lymphoma cells and cells of the microenvironment by taking part in the recruitment of distant innate immune cells known to support tumor growth. Hansen et al. reported that CD30+ EVs caused a CD30-dependent release of the tumorigenic chemokine interleukin-8 in CD30 ligand (+) eosinophil-like cells and primary granulocytes [84]. CD30 antibody constructs directed towards CD30+ EVs would be of considerable therapeutic efficacy in classical HL to block the CD30–CD30 ligand interaction in trans.

Lymphoma-derived EVs released by HL cells were also shown to carry high levels of the metallopeptidase ADAM10, a sheddase of CD30, TNFα, and the prototypical NKG2D ligand MICA. Shedding of ADAM10’s substrates is reported to hinder both anti-tumor immune responses and antibody-drug conjugate (ADC)-based immunotherapy. In a recent study, ADAM10 inhibitors were successfully used to inhibit ADAM10 activity in vesicles and prevent MICA shedding, leading to recognition of HL cells by cytotoxic lymphocytes [85]. EVs enriched in active ADAM10 can be considered not only as potential markers of the disease, but also a biomarker to monitor response of patients to immunotherapy. Personalized monitoring of EV-ADAM10 in circulation would be highly important in patients resistant to clinically used immunotherapeutic drugs, such as the ADC brentuximab vedotin or the anti-CD30 iratumumab, and the use of ADAM10 inhibitors may re-sensitize patients to antibody-based therapy.

Similar to what was shown in the hematological malignancies discussed in previous sections, a large number of miRNAs were highly enriched in EVs in patients with classical HL. Among more than 400 miRNAs identified, purified EV fractions carrying miR-*21*, miR-*155*, miR-*24-3p*, miR-*127-3p*, and let-*7a-5* were the most highly released in the sera of HL patients compared with EV fractions from healthy subjects and disease-free controls. In addition, the circulating levels of the aforementioned EV-miRNAs directly correlated with disease progression. Serial monitoring of those EV-miRNA levels in patients before treatment, directly after treatment, and during long-term follow-up revealed a decrease in EV-miRNA levels upon successful therapy, whereas their levels in serum rose again in patients who relapsed [86]. Monitoring plasma levels of the aforementioned EV-miRNAs could serve as an accurate measure of disease response easily quantifiable within the laboratory in order to assist optimal treatment for each HL patient.

The potential of lymphoma cell-derived EVs enriched in microRNAs as prognostic markers and predictors of treatment efficacy has also been demonstrated in patients with diffuse large B-cell lymphoma (DLBCL). Next-generation sequencing performed to identify EV-miRNA profiles from parental and chemoresistant lymphoma cells showed that, of 37 upregulated LCEV-microRNAs in circulation, LCEV-miRNA-*99a-5p* and miRNA-*125b-5p* levels were significantly increased and directly correlated with shorter progression-free survival and drug resistance [87].

In another interesting study, Provencio et al. evaluated the feasibility of known oncogenic mRNAs packed in EVs as a possible liquid biopsy method for the monitoring and prognostic evolution of B-cell lymphomas. Among the several EV-mRNAs tested in circulation (EV-c-MYC, BCL-XL, BCL-6, NF-κβ, PTEN, and AKT), high levels of EV-c-Myc and BCL-6 were independently correlated with shorter overall survival and worse progression free survival. Additionally, EV-c-Myc mRNA levels were selectively increased in the sera of patients that did not completely respond to first in-line therapy. High EV-c-Myc mRNA levels could serve as significant monitoring markers of poor prognosis and incomplete therapeutic response in patients with B-cell lymphomas [88].

Waldenström macroglobulinemia (WM) is a low-grade B-cell lymphoma characterized by disease progression from IgM MGUS to asymptomatic, and then symptomatic disease states. WM’s evolution and pathogenesis are still not clearly understood because of the long periods of asymptomatic disease states in some patients. Such a phenomenon may confuse clinical treatment decision-making. The miRNA content of circulating EVs was shown to be unique markers of WM disease evolution. A small group of EV-miRNAs were abundantly released in patients’ sera and correlated directly or inversely with disease status. The expression levels of the known tumor suppressor miRNA let-*7d* packed in EV were inversely correlated with disease stage, whereas EV-miR-*21*, as well as EV-miR-*192* and miR-*320b*, directly correlated with disease progression. More importantly, similar serum levels of the aforementioned miRNAs in EVs from patients with asymptomatic and symptomatic WM were observed as compared to disease-free control subjects, suggesting that the environmental and clonal changes occur in patients at early stages of the disease [89]. Changes in those EV-microRNAs serum levels showed to be robust predictors of disease progression and could be used as a liquid biopsy within the laboratory to guide clinicians for optimal diagnosis of the disease and to distinguish it from MGUS, especially in asymptomatic patients.

An overview of the role of hematological malignancy-derived EVs as biomarkers of disease and therapeutic targets based on their content is summarized in Table 1. 

### 2.2. EVs As Mediators of Cross-Talk Between Malignant and Immune Cells

#### 2.2.1. Role of EVs in Immune Cell Evasion

Beyond their functions as biomarkers of disease and therapeutic targets in hematological malignancies, EVs also participate in the cross-talk between malignant and immune cells. Depending on the type of cargoes carried in each malignancy, EVs released by tumor cells exert immunomodulatory activities resulting in the suppression of the immune response, a phenomenon that helps progression of the disease. In this section, we will review the role of malignant cell-derived EVs in influencing escape from immune surveillance in the tumor microenvironment in the most prevalent hematological malignancies.

In **AML**, as briefly discussed earlier, patients’ sera contained high levels of tumor-derived EVs enriched in the immune suppressor molecule TGF-β1 [28]. TGF-β1 impaired the function of NK cells, which are important players of the innate immune response. Activation of the SMAD pathway was shown to be responsible for the downregulation of NKG2D expression, an NK-activating receptor involved in initiating a cytotoxic and cytokine response. Downregulation of NKG2D resulted in impairment of NK-cell cytotoxic activity [34]. In line with these findings, Hong et al. demonstrated that AML-derived EVs appeared to reduce the efficacy of NK-92 immunotherapy by transporting the inhibitory ligand (TGF-β1) to NKG2D, a surface-activating receptor for NK-92. EV delivery of high amounts of TGF-β1 was shown to be partly responsible for loss of NKG2D expression through activation of TGFβRI/II and SMAD 2/3 phosphorylation pathways [35].

AML tumor escape from T-lymphocytes’ immune surveillance was also described. Leukemic cell-derived EVs showed an immunosuppressive effect on T-cells. For instance, the immunosuppressive protein programmed death-receptor ligand molecule 1 (PD-L1) on the surface of tumor cells binds its receptor PD-1 on effector T-cells, thereby suppressing their activity. Antibody blockade of PD-L1 can activate an anti-tumor immune response, and is used as an immune checkpoint inhibitor treatment in AML patients. AML-derived EVs contain high amounts of PD-L1. EV-enriched PD L1 were shown to sequester anti-PD-L1 antibodies, resulting in suppression of T-cell activity and resistance of AML cells to immune checkpoint inhibitors [90]. Moreover, removal of exosomal PD-L1 inhibited tumor growth in models resistant to anti-PD-L1 antibodies, and re-sensitized them to the immune checkpoint inhibitor treatment in an additive manner [91]. Taken together, these findings show that exosomal PD-L1 abundantly released by AML cells represents a potent immunosuppressor of T-lymphocytes.

**CML**-derived small EVs were also found to be typically enriched in TGF-β1, which has been shown to impact the immune system and induce tumor cell proliferation [92]. However, little is known about what cellular immune subtypes are altered by TGF-β1 and by which mechanism(s) TGF-β1 exerts its functions. More studies in CML are needed in this regard.

Other important immune cells present in the microenvironment that have been modified by tumor-derived EVs are macrophages. Macrophages have the plasticity to be polarized to either M1 tumoricidal or M2 tumorigenic macrophages. Under various stimuli, M2 macrophages are educated into tumor-associated-macrophages (TAM) known to release pro-tumorigenic growth factors, chemokines, and cytokines that support tumor development. CML-derived EVs have been shown to induce M2-like macrophage polarization, leading to the overexpression of the pro-tumorigenic cytokines IL-10 and TNF-α. They also caused a modification in the redox potential of macrophages that resulted in downregulation of the inducible nitric oxide synthase (iNOS), which in turn caused reduction of nitric oxide (NO) and reactive oxygen species (ROS) levels. A decrease in iNOS levels is a major modification responsible for re-educating macrophages towards TAM [93]. These results suggest that CML-derived EV alter the local bone marrow niche toward a leukemia-reinforcing microenvironment by skewing macrophage polarization towards a tumor-supportive phenotype.

Similar to macrophages, neutrophils also show phenotypic plasticity, which is modulated by different external signals, causing their polarizing to either pro- or anti-tumor neutrophils. CML-derived EVs affected polarization of neutrophils as well. As mentioned in earlier sections, BCR/ABL mRNA and protein are highly detected in EVs of CML patients. Other than its role in CML diagnosis, EVs carrying BCR/ABL were shown to be transferred to neutrophils both in vitro and in vivo, causing aberrant gene expression in the target cells that resulted in neutrophil skewing towards tumor-associated neutrophils. Tumor-associated neutrophils showed low cytotoxicity against CML cells and recapitulated CML-like symptoms when injected in mice [94].

In **CLL**, the T-cell compartment was the most affected by circulating EVs. CD4+ T-cells isolated from CLL patients have been shown to internalize CLL-derived small EVs containing high levels of onco-miR-*363*, whose levels were shown to correlate with the CLL disease stage. MiR-*363* was demonstrated to directly target the immunomodulatory receptor CD69 [95]. CD69 expressed on the surface of T-cells is an early T-cell activation molecule known to act as a co-stimulatory receptor [96]. Other important immune cells that play an essential role in immune escape are monocytes. Under inflammatory conditions, circulating monocytes are recruited to the inflammatory sites and can be differentiated into either macrophages or dendritic cells under different stimulus factors. However, in cancer, monocyte plasticity is reduced by tumor-derived EVs, leading them to reside in the tumor microenvironment in an immature state. Indeed, a recent study has evaluated the role of CLL cell-derived EVs in the cross-talk with monocytes. Haderk et al. showed that EVs derived from the plasma of CLL patients carry high amounts of the non-coding RNA hY4 with respect to disease-free control samples. EV-hY4 transferred to monocytes contributed to their undifferentiated state. Immature monocytes induced release of key CLL-associated pro-tumorigenic chemokines and cytokines, such as CCL2, CCL4, and interleukin-6, as well as the T-cell immunosuppressor PD-L1, whereas in vivo blocking of the EV-hY4 pathway in CLL-bearing mice decreased leukemia development [97]. These findings showed how, by communicating with monocytes, CLL-derived EVs indirectly impaired the immune system and contributed to CLL tolerogenic inflammation.

In **MM**, NK cells are the cell type that is the most targeted by MM-derived EVs. Several studies have repeatedly shown that MM-derived small EVs significantly reduce the cytotoxic activity of NK cells against MM cells [98,99,100]. The reason for the strong inhibition of NK-cell activity is that MM-derived EVs induce a significant decline in the expression levels of not only one, but most of the activating receptors known to be essential in inducing NK cytotoxicity, including NKp46, NKp30, and NKG2D [98]. Another study went even further and analyzed the effect of MM-derived EVs on NK cells under genotoxic stress. Although anti-cancer chemotherapeutic agents were traditionally known to enhance the immunogenic potential of malignant cells, this concept is no longer widely accepted. Vulpis et al. demonstrated that when MM cells are exposed to sublethal doses of melphalan, a genotoxic drug currently used in the clinical management of MM patients, a spike in EV release was observed. MM-derived EVs taken up by NK cells are capable of stimulating IFNγ production, but not their cytotoxic activity, through a mechanism based on the activation of the NF-κB pathway in a TLR2/HSP70-dependent manner [100]. The reason for such an increase in IFNγ production by NK cells but not in their activity is that under genotoxic stress, MM cells were observed to release high amounts of the metalloproteinase ADAM10, known to shed NKG2D receptor ligand MIC. Once shed, NK receptor ligands become soluble and act differently than membrane-associated receptors by blocking the receptors, instead of activating them. As a result, NK cells lose their capacity to secrete another important cytokine, TNFα, that is known to stimulate their cytolytic activity [101]. 

Another MM-EV cargo that strongly suppresses NK-cell function is the MM-associated antigen, CD38. As mentioned earlier, MM-derived EVs release high amounts of the soluble ectoenzyme CD38 [36]. CD38 has been suggested to convert nucleotides to adenosine, leading to an anergic immune response. Adenosine binds to the purinergic P2 receptors present on the surface of immune cells to result in the anergic status of T-cells, dendritic cells, and, to a large extent, NK cells [102].

Other components of the tumor microenvironment that play an essential role in immune escape are myeloid-derived suppressor cells (MDSCs). MDSCs are a small population of immature myeloid cells that accumulate in the MM bone marrow microenvironment and suppress the function of multiple immune effector cells, particularly T-cells. Other than MM-derived EVs, EVs produced by MM-stromal cells have also been reported to impede the immune response. BMSC-derived EVs taken up by MM-MDSCs are able to induce the expansion of the latter through activation of STAT3 and STAT1 pathways and by increasing the expression of the anti-apoptotic proteins, Bcl-xL and Mcl-1. These EVs also induce an increase in nitric oxide release from MDSC, which in turn suppresses T-cells immune reaction, favoring MM cell survival [103].

Similar to the hematological malignant cells discussed above, **lymphoma** cells use the potential of EVs to diminish the overall immune response. Reports have shown that lymphoma-derived EVs mainly target T-cells and MDSCs to impede immune surveillance. Indeed, Chen et al. have demonstrated that diffuse large B-cell lymphoma-derived EVs are rapidly captured by T-cells and upregulate the inhibitory receptors PD-1, CTLA-4, and BTLA, leading to apoptosis of T-cells through activation of the Fas/FasL pathway and resulting in tumor growth in vivo [104]. In a recent study, Zhang et al. also showed that tumor B-cell-derived small EVs enriched with the ectonucleotidases CD39 and CD73 were able to hydrolyze ATP into adenosine, which is known to affect the cancer immune response, causing inhibition of post-chemotherapeutic CD8+ T-cell proliferation and cytotoxicity. Moreover, silencing of EVs released from tumor B-cells significantly improved the antitumor effect of chemotherapy and CD8+ T-cell responses in vivo [105].

MDSCs are also affected by lymphoma-derived EVs. HSP72 expressed on the surface of lymphoma-derived EVs have been shown to promote T-cell suppression functions of MDSCs via activation of STAT3 in a TLR2/MyD88-IL6-dependent manner. Mechanistically, Hsp72 triggers the TLR2 signaling pathway within MDSCs, which leads to an autocrine IL-6 secretion. In turn, IL-6 induces STAT3 phosphorylation, which was shown to be responsible for the immunosuppressive activity of MDSCs on T-cells [83]. In aggressive B-cell lymphoma, which includes DLBCL, Burkitt lymphoma, mantle cell lymphoma, and B lymphoblastic lymphoma, humoral immunotherapy is largely used in clinics because of the aggressiveness of the disease and its resistance to conventional chemotherapy. However, patients’ responses to treatment varies, with a majority of them developing resistance to anti-CD20 antibody therapy. A study using patients’ samples demonstrated that aggressive B lymphoma cells release large amounts of the tumor-related antigen CD20 in circulation and shield target cells from antibody attack, leading to B-cell lymphoma escape from antibody-dependent cell-mediated cytotoxicity and complement-dependent cytotoxicity. The mechanism of EV release in circulation was seen to be modulated by the ATP-binding cassette transporter A3. Indeed, both pharmacological blockade and the silencing of ABCA3 enhanced the susceptibility of lymphoma cells to antibody-mediated lysis [82]. In this specific type of lymphoma, inhibition of EV biogenesis or release may be an effective procedure for improving patients’ responses to antibody therapy.

An overview of the role of hematological malignancy-derived EVs on immune escape based on their content and the type of target cell is summarized in Table 2. 

#### 2.2.2. Role of EVs in Anti-Tumor Immunity and Immunotherapy 

In hematological malignancies, the dual role of tumor-derived EVs in modulating anti-tumor immunity is still under investigation. However, there are enough data showing that, by manipulating tumor-derived EVs and their interaction with immune cells, the immune system regains its normal functions to eradicate tumors. One major focus in the field is to target dendritic cells (DCs) by modified secretory EV in order to reinitiate T-cell immunity. This approach is based on the biology of DCs as antigen-presenting cells. EVs carrying tumor-specific antigens may be taken up by DCs and loaded on their MHCs. In turn, DCs present the antigens and prime tumor-specific T lymphocytes to reactivate the immune response.

For instance, treatment of DCs with tumor-derived EVs induced strong specific antileukemic immunity. EVs derived from **CML** cells were pulsed on DCs and resulted in a robust antileukemic T-cell immune response, as well as protective immunity against leukemic cells, both in vitro and in vivo. Significantly prolonged survival was also observed in leukemic mice immunized with CML-EVs pulsed DCs, compared to ones injected with non-pulsed DCs. Interestingly, on days 7–10 after immunization, all mice injected with non-pulsed DCs showed tumor growth, whereas 87.5% of CML-EV-immunized mice were tumor-free [106]. Moreover, an exosome-based vaccine was also effective in promyelocytic leukemia. A cytotoxicity assay demonstrated that cytotoxic T lymphocytes (CTLs) induced by DCs pulsed with Hsp70+ vesicles derived from a human promyelocytic leukemia cell line were significantly more effective in killing target leukemic cells than CTLs induced by DCs alone [107]. EVs were also designed as potent vaccine adjuvants and immune regulators. Vesicle-pulsed DCs not only augmented the specific T-cell response, but also gave rise to a Th1-type shift and an antibody response. Qazi et al. showed that EV-pulsed DCs provided antigens for B-cell activation. B-cell activation was necessary for EV-based T-cell stimulation, as blocking B-cell differentiation at its early stages showed abrogated B- and T-cell responses following EV immunization [108]. In addition to the effective anti-tumor activity of EV-pulsed DCs, engineered EVs were also applied as useful therapeutic tools. CML cell-derived EV decorated with the death ligand TNF-Related Apoptosis-Inducing Ligand (TRAIL) infiltrated lymphoma and MM cells and induced apoptosis in those cells. In addition, systemic administration of TRAIL (+) EVs in vivo accumulated in the liver, lungs, and spleen, and homed to the lymphoma tumor site, leading to a significant reduction of tumor growth [68].

In **AML**, an EV-DC vaccine showed a dual effect. In preclinical studies, AML-derived EVs successfully pulsed DCs and activated a T-cell immune response, resulting in lysis of leukemic cells, both in culture using AML cell lines and in vivo by using an engrafted murine model of AML [109,110]. However, in an attempt to activate the antigen-presenting capacity of DCs using EVs from human primary samples as an antigenic source, incubation of purified EV from AML patients with DCs, T lymphocytes, and leukemic cells showed a decrease in IFNγ production by effector T-cells, resulting in decreased lysis of target leukemic cells, as well as downregulation of the DC costimulatory molecule CD86 [109]. This phenomenon may correspond to a mechanism of immune system evasion, instead of activation. The discrepancy between preclinical studies and studies using patients’ samples may be because, for one, the tumors in mice were not autologous but transplanted, and the immune system in mice does not reflect the immunosuppressive microenvironment present in AML patients. Second, cultured AML cell lines may be more prone to releasing pure tumor-specific antigens for DC activation, whereas circulating EV isolated from patients may contain larger amounts of immunosuppressive molecules that do not necessarily originate from AML blasts, but other compartments in the tumor microenvironment, thus driving DCs toward a suppressive phenotype. More studies are needed in this regard to identify specific tumor-associated antigens for successful development of a DC vaccine in AML.

As mentioned earlier in this review, EVs enriched in TGFβ1 exert a strong immunosuppressive function, both in AML and CML. On the basis of those findings, in an interesting study, Huang et al. attempted to specifically manipulate leukemic cells in order to enhance the efficacy of an EV-DC-based vaccine. They showed that EVs derived from TGF-β1-silenced leukemic cells promoted DC maturation and subsequent T-cell proliferation, Th1 cytokine secretion, and a tumor-specific CTL response [111]. Priming DCs with modified EVs by knocking down potent immunosuppressors could be a useful approach to improve DC vaccine therapy in leukemia.

Another potential immunotherapeutic application of EV is their use as agents to restore NK-cell activity. For instance, the soluble ligand of the activating receptor Nkp30 (BAG6), highly detectable in the sera of **CLL** patients at advanced disease stages, was shown to suppress NK-cell cytotoxicity. However, BAG6 presented on the surface of vesicles triggered NK cytotoxicity, whereas exosomal BAG6-deficient leukemic cells evaded NK-cell immune surveillance [112]. 

Another aspect of CLL cells that makes them suitable hosts for EV engineering therapy is that they express high levels of surface MHC class I and II molecules, but lack expression of important accessory and co-stimulatory molecules, making them potential, but non-functional antigen-presenting cells. In a recent study, Gartner et al. developed a novel immunotherapeutic approach to re-initiate the antigen-presenting capacity of CLL cells based on engineered EVs. Treatment of primary CLL cells with EVs carrying the major glycoprotein of the Epstein bar virus (EBV) (gp350), the central immune accessory molecule (CD40 ligand), and the immunodominant antigen of the human cytomegalovirus (CMV) (pp65) restored their antigen-presenting capacity and rendered them immunogenic to allogenic and autologous EBV- and CMV-specific CD4+ and CD8+ T-cells [113]. This is the most recent, and maybe the only study that used EVs as therapeutic tools to re-target the strong herpes viral immunity in CLL patients to malignant cells. This approach could constitute an attractive strategy for adjuvant treatment of CLL.

In **MM**, the role of EVs as possible tools in immunotherapy has also been investigated. Studies have shown successful results in priming T-cells, as well as in re-initiating NK cytotoxicity. Leaf et al. developed a novel potential anti-myeloma allogenic vaccine using a human myeloid leukemia cell line differentiated into a fully functional dendritic cell, known as the DCOne vaccine. The potential of this cell line is that, when pulsed with MM-derived EVs, it produces a vast range of MM-specific antigens encapsulated in small EVs. Co-culture of patients’ plasma cells with peripheral blood mononuclear cells and DCOne induced a strong expansion and activation of cytotoxic T lymphocytes, resulting in the killing of autologous MM cells [114]. This study shows that allogenic DCOne is an improved version of a DC vaccine, overcoming the complications of primary dendritic cell functions. The DCOne vaccine could be a more advanced tool to reactivate T-cell immune responses, not only in MM but possibly also in other hematological malignancies. Although treatment of MM with genotoxic drugs, such as doxorubicin or melphalan, were shown to enhance EV release and immunosuppression [100], for the first time, a recent study revealed one way of overcoming MM-EV suppression of the NK immune response by reactivating the IL-15 signaling pathway. Upon stimulation with melphalan, MM cells and MM-derived EVs showed increased expression of the IL-15 receptor A/IL-15 ligand complex. When melphalan-treated, MM-derived EVs were incubated with NK cells in the presence of exogenous IL-15, an increase in NK-cell proliferation and killing of MM cells were observed [115]. This report demonstrates the potential of the IL-15 signaling pathway as a strong activator of NK-cell immunity regardless of the many suppressor factors present in the MM microenvironment.

Manipulating circulating EVs to reactivate anti-tumor immunity was also described in several models of **lymphoma**. The oncoprotein c-Myc plays an essential role in B-cell malignancies. EVs have been evaluated as drug delivery vehicles to deactivate c-Myc downstream functions. Vesicles carrying the c-Myc silencing RNA complex were successfully delivered to lymphoma cells and reduced endogenous c-Myc transcripts, as well as its downstream targets, resulting in lymphoma cell death [116]. An EV-DC vaccine was also effectively evaluated to re-activate immune surveillance in lymphoma. Unlike what was observed when DLBCL-derived EVs were directly incubated with T-cells and induced their inhibition by secreting potent immunosuppressive molecules [104], DLBCL-derived small EVs carrying tumor-specific antigens pulsed into DCs reactivated the antigen-presenting capacity of DCs, resulting in a cross-priming of CD8+ T-cells and a potent CTL-dependent lysis of syngeneic and allogeneic tumors [117].

## 3. Isolation and Characterization of EVs for Liquid Biopsy

EVs are abundantly present in biofluids. Plasma and serum are the most common body fluids used for liquid biopsies in hematological malignancies because they are easy to collect and peripheral blood is the primary environment in which EVs are released [118]. Several methods have been implied to isolate EVs in this context. However, the field has not yet reached the optimal technique for isolating ultrapure EVs because of the presence of contaminant glycoproteins, protein-nucleic acid aggregates, and glycolipids in plasma that can affect EVs’ recovery; thus, accurate analysis of data in the context of liquid biopsy is still a challenging task [119].

(i) Ultracentrifugation is the most common method of isolation of EVs from plasma or serum [120]. Because their size is in the magnitude of viruses, EVs can form a pellet at the bottom of an ultracentrifuge tube upon applying a high-speed centrifugal force. Two types of ultracentrifugation techniques have been described on the basis of the methods of separation: differential centrifugation and density gradient centrifugation. Differential centrifugation is based solely on centrifugation force, whereas density gradient centrifugation separate EVs on the basis of their mass density using a density gradient medium, most commonly sucrose [121]. Differential centrifugation is routinely applied to isolate plasma or serum EVs. Although it is easy to perform, because plasma contains a complex mixture of many components, including vesicles of different sizes and protein complexes, by only applying a centrifugal force as a means of separation, isolation of pure EVs free of contaminants is impossible. As a result, EVs tend to form aggregates of varying sizes with different protein complexes [119]. To separate plasma contaminant protein complexes from EVs, Muller et al. added density gradient centrifugation following differential centrifugation, using sucrose as a density gradient medium. Contaminant proteins accumulate into different density layers. EV purity was significantly improved, but was not fully optimal for biomarker analysis [120]. (ii) Ultrafiltration is another method of EV separation. It relies on the use of membranes with specific pore sizes to eliminate large particles, leaving a relatively concentrated filtrate of EVs [122]. Ultrafiltration is commonly used for EV isolation from cell culture supernatants, especially when using suspended cells grown in a large volume of media. It allows researchers to concentrate the vesicular population for improved characterization. Because culture media is considered a relatively simple chemical composition devoid of protein or sugar contaminants, EV purity is not an issue in this case. We have routinely used ultrafiltration as a method of EV extraction from cultured MM cells for biomarker detection or functional analysis [67,76]. Although isolation of urinary EVs using a nanomembrane ultrafiltration concentrator has been shown to yield a high purity index suitable for the analysis of exosomal biomarkers [123], ultrafiltration is not the primary method applied to isolate EVs from plasma or serum because of significant losses due to unspecific membrane adsorption and the limited amount of blood available from cancer patients to use for liquid biopsy. However, it can be used as a second step after ultracentrifugation to increase the purity of EVs [120]. (iii) Precipitation is another separation technique that uses a poly-ethyl glycol aqueous solution to coat EVs, which in turn can aggregate and precipitate by low-speed centrifugation [124]. Several kits have been developed and optimized in order to isolate EVs from plasma or serum. Exoquick™ (System Biosciences) and Exo-spin™ (Cell Guidance Systems) were tested to isolate plasma EVs for the purpose of use as prognostic and diagnostic markers. Although both Exoquick™ and Exo-spin™ gave a high yield of EV recovery, exosomal purity and specificity were poor because of co-precipitation of non-exosomal proteins, including a high degree of albumin contamination [125]. (iv) Size Exclusion Chromatography (SEC) is the most advanced separation technique based on size that has been recently adapted for the separation of EVs. It uses the biofluid as a mobile phase and a porous gel filtration polymer packed in a column as the stationary phase. Briefly, large particles (usually protein contaminants) are the first to be eluted because of the fewer pores they can traverse, while smaller particles (EVs) are eluted last [126]. In attempts to optimize the quality and purity of EVs’ isolation from plasma and serum for diagnostic and functional purposes, SEC has been reproducibly shown to outperform several other separation methods [120,125,127]. It provided the highest degree of EV purification from protein contaminants, even though the recovery yield was somewhat lower than what was observed using other separation techniques [125]. Interestingly, SEC has been regularly applied as a final stage for the isolation of EVs from plasma and serum, a “cleaning” step for the characterization of EV cargoes in hematological malignancies using high-throughput-based technology [118]. A list of the methods commonly used for the isolation and purification of plasma and serum EVs in hematological malignancies is summarized in Table 3.

Several attempts have been made to optimize the best approach to separate EVs from plasma or serum using a combination of the aforementioned techniques for the most accurate analysis of disease biomarkers. So far, the most up-to-date method was demonstrated by Muller et al. This research group showed that the application of SEC following differential centrifugation and ultrafiltration resulted in high purity and superb recovery of morphologically and functionally intact EVs suitable for high-throughput cargo characterization in the context of liquid biopsy [118,122]. 

The characterization of EVs depends on the type of cargo. As mentioned earlier in this review, EV cargoes that were used as biomarkers of disease in hematological malignancies are mostly either of RNA or protein origin. For EV cargoes of RNA origin (either mRNA or microRNA), the most common accurate biochemical analytical method used in this context is real-time polymerase chain reaction following total RNA extraction from isolated EVs [67]. As for EV cargoes of protein origin, the classical biochemical analyses used in this context are mainly immunoblotting and immunosorbent assays, as well as an indirect optical detection method, flow cytometry, which determines surface protein expression on exosomes [128]. However, in view of the need of highly pure EVs for accurate analysis of disease biomarkers and the limited amount of plasma or serum than one can obtain from patients for this purpose, high-throughput analysis is becoming the most developed and commonly used method for the characterization of EVs. High-throughput technologies are automated technologies, including mass spectrometry-based approaches, next-generation sequencing, and microarrays, among others, that permit a thorough characterization of the content of EVs while using a minimum amount of starting material [127]. For instance, in order to define specific circulating myeloma-associated protein markers, we have used global systemic proteomic analysis as the main characterization method of EVs isolated from MM patients’ serum. We have also adapted the same screening method to identify a prognostic marker in corticosteroid-resistant MM [76]. In another study, Hornick et al. performed a comparative microarray analysis to identify a specific serum exosomal microRNA signature that could serve as a clinical prognostic tool for the detection of AML [40].

## 4. Conclusions

**Figure 1:**
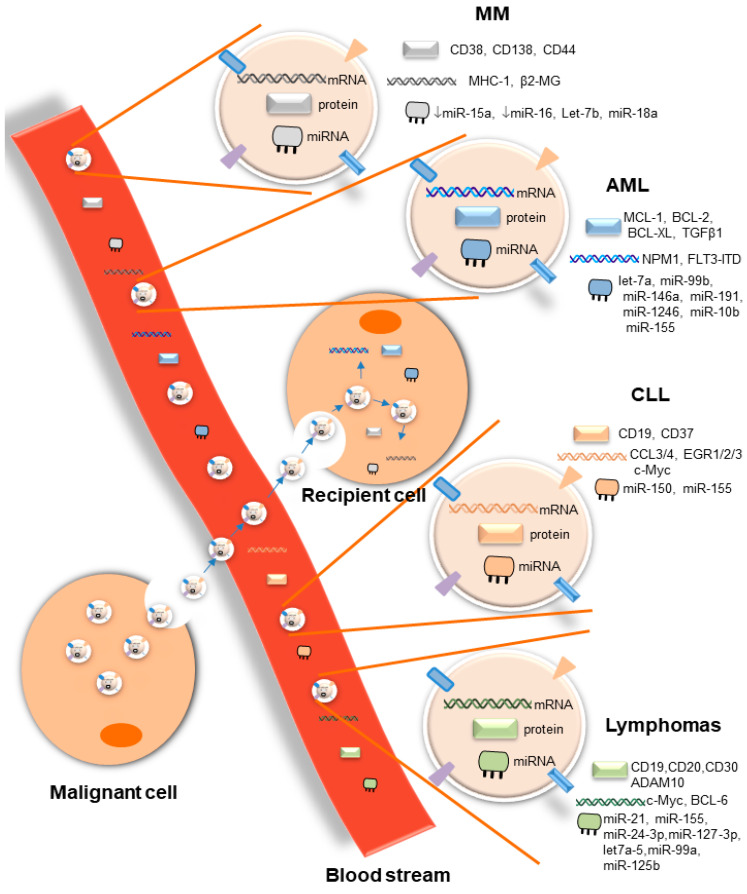
Diagram showing circulating EVs released from a malignant cell to the bloodstream containing bioactive molecules (proteins, mRNAs, microRNAs) that serve as both biomarkers of disease and therapeutic targets in the most prevalent hematological malignancies: MM, AML, CLL, and lymphomas. Once in the blood, EVs either release their contents or mediate a cell–cell communication at a distant site, where they are taken up by a recipient cell. The internalized EVs discharge their cargos, which induce changes in the physiological landscape of the recipient cell, resulting in tumor growth and/or drug resistance. The recipient cell could be either a hematopoietic cell or a cell of the bone marrow microenvironment.

EVs are important mediators of cell–cell communication at short and long distances, both in health and disease. In hematological malignancies, through the release of small EVs, malignant cells interact with a large number of cells, including cells of the tumor microenvironment that substantially support disease progression by inducing tumor drug resistance and immune suppression. EVs released by malignant cells or cells of the microenvironment contain a large repertoire of tumor-specific cargoes, including antigens, oncogenes, and onco-microRNAs, among others, as summarized in this review (Figure 1). This considerable range of tumor-specific EVs that reflects the cell of origin makes them potential therapeutic targets. However, broad suppression of EV biogenesis or release may be a double-edged sword, given the critical role of EVs released by healthy cells in maintaining normal physiological responses [129]. For that reason, advanced technologies for EV-selective and specific targeting based on the molecular properties of their cargoes are absolutely needed. Although the molecular mechanisms involved in EV biogenesis and release have been described, the identity of vehicle surface molecules involved in guiding the route of EVs to specific target cells will be critical for the development of selective targeted therapies for EVs. In addition, tumor-derived EVs are highly abundant in biofluids, and their content is completely different from that of EVs released by non-cancerous cells. These unique features seen in several hematological malignancies make them potential biomarkers for diagnosis, prognosis, disease progression, and therapy resistance. Although pre-clinical studies have shown the importance of EVs in the context of liquid biopsies of hematological malignancies, veritable clinical applications are waiting for validation in large prospective clinical trials. The potential uses of EVs have been confirmed in several phase I and II clinical trials in solid tumors [130,131,132]. However, no single clinical trial has been conducted so far in blood cancer. In light of the importance of liquid biopsies in hematological malignancies, initiation of clinical trials of the prospective uses of EVs is an urgent need.

Overall, several questions remain to be answered in the field of EVs. One major challenge is to better understand the in vivo impact of tumor-derived EVs on the different components of the host immune system relevant to cancer progression, as well as the molecular mechanisms responsible for target cell specificity and routes of cellular uptake.

## Figures and Tables

**Table 1:** Overview of the hematological malignancy-derived EVs as potential biomarkers of disease and therapeutic targets on the basis of their content.

Hematological Malignancies	EV Content	Biomarker Use	Therapeutic Targets	References
AML	NPM1, FLT3-ITD mRNAs	Prognosis	yes	[31]
	MCL-1, BCL-2, BCL-XL	Drug resistance	yes	[32]
	CXCR4, IGF-IR mRNAs	No	yes	[31]
	CD33, CD34, CD117	Diagnosis	NI	[34]
	TGFβ1	Diagnosis, drug resistance	yes	[34]
	CD13	Disease progression	NI	[36]
	let-*7a*, miR-*99b*, miR-*146a*, miR-*191*, miR-*1246*, miR-*10b*	Diagnosis, Prognosis	yes	[41,42]
	miR-*155*	Diagnosis, prognosis, drug resistance	yes	[40,42,44,45]
CML	BCR/ABL mRNA	Diagnosis	yes	[47,48]
	TGFβ1	Prognosis	yes	[49]
	miR-*92a*	No	yes	[52]
	↓ miR-*215*	UMRD upon imatinib discontinuation	No	[53]
CLL	CD5, CD31, CD44, CD55, CD62L, CD82, HLA-A, HLA-B, HLA-C, HLA-DR	Diagnosis	NI	[58]
	CD19, CD37	Disease progression	yes	[59]
	CD52	Disease progression, drug resistance	no	[60]
CLL	miR-*150*, miR-*155*	Diagnosis, Prognosis	yes	[61,62]
	CCL3/4, EGR1/2/3, c-Myc	Drug resistance	yes	[57]
	miR-19b	Diagnosis of RS, multidrug therapy resistance	yes	[63]
MM	↓ miR-*15a* ↓ miR-*16*	Prognosis, Drug resistance	no	[65,67,73,74]
	LNC00461	NI	yes	[69]
	miR-*146a*	NI	yes	[70]
	Let-*7b*, miR-*18a*	Diagnosis, Prognosis	yes	[72]
	CD38, CD138, CD44	Prognosis, Disease progression	yes	[36,75,76]
	MHC-1, β2-MG	Diagnosis	yes	[76]
	CD46, CD26, ↓ CD31,↓ CD106	GVHD	NI	[79]
	CD138-P-gp+CD34+	Multidrug resistance	NI	[80]
NHL	CD19, CD20	Diagnosis, Prognosis, Disease progression antibody-therapy resistance	yes	[36,82]
HL	CD30	Diagnosis, Prognosis	yes	[81]
	ADAM10	Antibody-therapy resistance	yes	[85]
	miR-*21*, miR-*155*, miR-*24-3p*, miR-*127-3p*, let-*7a-5*	Prognosis, Disease progression	yes	[86]
DLBCL	miR-*99a-5p*, miR-*125b-5p*	Prognosis, Drug resistance	NI	[87]
	c-Myc, BCL-6 mRNAs	Prognosis, Drug resistance	yes	[88]
WM	miR-*192*, miR-*320b* ↓ let-*7d*	Diagnosis, Disease progression	NI	[89]

Abbreviations: Acute Myeloid Leukemia (AML); Chronic Myeloid Leukemia (CML); Chronic Lymphoblastic Leukemia (CLL); Multiple Myeloma (MM); Non-Hodgkin’s Lymphoma (NHL); Hodgkin’s Lymphoma (HL); Diffuse Large B-Cell Lymphoma (DLBCL); Richter Syndrome (RS); Undetectable Minimal Residual Disease (UMRD); Waldenström Macroglobulinemia (WM); Graft Versus Host Disease (GVHD); ↓ (low levels); Not Identified (NI).

**Table 2:** Overview of the role of hematological malignancy-derived EVs on immune escape based on their content and type of target cell.

Hematological Malignancies	EV Content	Target Cells	Effects	References
AML	TGFβ1	NK cells	↓ NKG2D impairment of cytotoxicity	[34,35]
	PD-L1	CD8+ T-cells	Impairment of cytotoxicity	[90]
CML	NI	Macrophages	↓ iNOS ↑ TAM	[93]
	BCR/ABL	Neutrophils	↑ TAN recapitulate CML-like symptoms	[94]
CLL	miR-*363*	CD4+ T-cells	↓ co-stimulatory receptor CD69	[95,96]
	non-coding RNA hY4	Monocytes	Contribution to undifferentiation ↑ CCL2, CCL4, IL-6, PD-L1	[97]
MM	ADAM10, MIC, CD38	NK cells	↓ NKp46, NKp30, NKG2D Anergic status Impairment of cytotoxicity	[36,98,99,100,101,102]
	BMSC-EV (content NI)	MDSC	↑ MDSC proliferation ↑ NO ↑ T-cell death	[103]
DLBCL	c-Myc, Bcl-2, Mcl-1, CD19, CD20	T cells	↑ PD-1, CTLA-4, BTLA Apoptosis	[104]
BL	CD39, CD73	CD8+ T-cells	Impairment of proliferation and cytotoxicity	[105]
T-cell lymphoma	Hsp72	MDSC	↓ IFN-γ production by T-cells	[83]

Abbreviations: Acute Myeloid Leukemia (AML); Chronic Myeloid Leukemia (CML); Chronic Lymphoblastic Leukemia (CLL); Multiple Myeloma (MM); Diffuse Large B-Cell Lymphoma (DLBCL); Burkitt Lymphoma (BL); Myeloid Derived Suppressor Cells (MDSC); Tumor-Associated Macrophages (TAM); Tumor-Associated Neutrophils (TAN); ↓ (decrease); ↑ (increase); Not Identified (NI).

**Table 3:** Summary of the methods used for isolation and purification of plasma and serum EVs in the context of liquid biopsy.

Method		Advantages	Limitations
Ultracentrifugation	Differential centrifugation	Commonly and routinely applied Method protocol well established [120,121]	Long and challenging procedure High rate of contamination with plasma protein complexes [119,121]
	Density gradient centrifugation	Commonly used Higher purity than differential centrifugation [120,121]	Difficult procedure Purity improved but not suitable for biomarker detection [120,121]
Ultrafiltration		High purity rate used for cleaning up before SEC [120]	Not commonly used Frequent loss of recovered material [119,120]
Precipitation		High recovery yield [125]	Low purity rate due to protein contamination [125]
SEC		High quality and purity rate Outperforms other methods for useful detection of disease biomarkers Used as a final “cleaning” step for accurate biomarker detection in hematological malignancies [118,120,125,127]	Relatively lower recovery yield [125]

Abbreviations: Size-Exclusion Chromatography (SEC).

## Abbreviations

EV	Extracellular Vesicle
MM	Multiple Myeloma
AML	Acute Myeloid Leukemia
CLL	Chronic Lymphoblastic Leukemia
